# Assessment of the Anterior Loop and Pattern of Entry of Mental Nerve Into the Mental Foramen: A Radiographic Study of Panoramic Images

**DOI:** 10.7759/cureus.55600

**Published:** 2024-03-05

**Authors:** Divakar Thiruvenkata Krishnan, Kingshika Joylin, Packiaraj I, Kandasamy M, John Hearty Deepak, Saraswathi Ilango, Khalid Al Hamad, Hanan Shanab, Mohammed Helmy Salama, Saikarthik Jayakumar

**Affiliations:** 1 Department of Oral and Maxillofacial Surgery, Rajas Dental College and Hospital, Tirunelveli, IND; 2 Department of Oral Medicine and Radiology, Rajas Dental College and Hospital, Tirunelveli, IND; 3 Department of Physiology, Madha Dental College and Hospital, Chennai, IND; 4 Department of Maxillofacial Surgery and Diagnostic Sciences, Majmaah University, Az Zulfi, SAU; 5 Department of Preventive Dental Sciences, Majmaah University, Az Zulfi, SAU; 6 Department of Maxillofacial Surgery and Diagnostic Sciences, College of Dentistry, Majmaah University, Al Majmaah, SAU

**Keywords:** radiography, opg, mandible, looping pattern, mental nerve

## Abstract

Introduction: The precise location of the mental foramina is an essential landmark in planning the position of dental implants in the anterior mandible. Injury to inferior alveolar nerve during anterior mandibular implant surgery causes altered sensation which greatly affects patient satisfaction.

Methods: In this study, we assessed the prevalence of anterior loop of mental nerve and the pattern of entry of mental nerve into the mental foramen. Three hundred panoramic radiographs (600 hemimandibles) obtained from records maintained in the Department of Oral Medicine and Radiology were randomly selected for the study. The radiographs were evaluated by two independent observers for the pattern of entry of mental nerve into the mental foramen on either side of the mandible and for the presence or absence of anterior loop of mental nerve.

Results: The most prevalent pattern of mental nerve observed was Straight pattern which totals to 67.5% followed by Anterior loop pattern (18.8%) and then the Perpendicular pattern (13.7%). There was no significant association between the gender and subtypes of looping pattern on the left and right side and a highly significant association between the side of the mandible and loop pattern was observed by Chi square test.

Conclusion: The Anterior loop pattern of mental nerve has been found in 18.8% of the population suggesting to accurate planning with three-dimensional imaging techniques to avoid injury to mental nerve during dental implant placement and other surgical procedure involving the interforaminal region of the mandible.

## Introduction

The inferior alveolar nerve, a branch of mandibular nerve enters the mandibular ramus at the medial aspect and runs down in the mandibular canal, supplying the mandibular teeth and the associated soft tissue structures. The mental nerve, one of the terminal branches of the inferior alveolar nerve, emerges through the mental foramen to supply the skin and mucous membrane of the buccal vestibule of the lower jaw from the medial border of the masseter muscle to the midline. Though a few studies have addressed the actual pattern of entry of mental neurovascular bundle into the mental foramen, the emphasis of most studies has been on determining the position of the mental foramen [1]. Anterior loop referred to inferior alveolar nerve where it passes backward, outward and upward to open at the mental foramen. A more precise description was reported by Bavitz et al., 1993, who described the anterior loop as the structure “where the mental neurovascular bundle crosses inferior and anterior to the mental foramen then doubles or loops back to exit the mental foramen [2]. This anterior loop is frequently identified in radiographs (11-60%) and hence posses clinical challenge for implant placement [3]. It has been evidenced in the literature that various pattern of entry of mental nerve have been noticed including straight, perpendicular and loop pattern [4].

The precise location of the mental foramina is an essential landmark in planning the position of dental implants in the anterior mandible [5]. The posterior-most implant of the anterior mandible is placed as close to the mental foramina as possible [6]. The prevalence of anterior loop of the inferior alveolar nerve (IAN) in the Indian population varies in literature and is around 56% with different studies stating different side predomination [7-9]. The anterior loop and other patterns of mental nerve plays a vital role in dental implants planning and in surgeries performed near the mental foramen. Before planning any surgical procedures of the anterior mandible, it is critical to consider this crucial anatomical variation [10]. Endosseous dental implant placements, bone harvesting, and osteotomy are some of the elective surgical procedures performed in anterior mandible which are deemed safe. However, one of the most bothersome complication of implant surgery in anterior mandible is post-operative altered sensation such as dysesthesia, paraesthesia, and hypoesthesia associated with injury to inferior alveolar nerve and its mental branch [11]. These post-operative complication even the milder ones like numbness or tingling drastically impacts patients’ satisfaction and may even result in medicolegal issues [12,13]. Short term and long term incidence of post-operative complication of altered sensation following anterior mandibular implant surgery were 13% and 3% respectively and 9% reported non regression of altered sensation even after one year [14]. In lieu of these potential debilitating complications, pre-operative radiographic examination of the mandible is essential and panoramic radiographs continue to be the widely employed radiologic diagnostic method for planning mandibular surgeries [6]. Hence in this study, we aimed to assess the prevalence of anterior loop of mental nerve and to assess the pattern of entry of mental nerve into the mental foramen.

## Materials and methods

Study design

The study was conducted based on the STrengthening the Reporting of OBservational studies in Epidemiology (STROBE) guidelines for cross-sectional studies [15]. The sample size was calculated using online software (Qualtrics.com) with 95% confidence interval, and with a margin of error at 5%. The required sample size for this study was around 370. A total of 387 radiographs were examined from 19th August 2022 to 18th October 2022 retrospectively. The entry pattern of the mental nerve into the mental foramen on either side of the mandible was evaluated by two independent observers in the radiograph.

Radiographic assessment

The Orthophos XG5 Sirona panoramic machine (Dentsply Sirona, Charlotte, NC) with its SIDEXIS XG viewing software were used for evaluation of digital panoramic images. The digital images were obtained from the Department of Oral Medicine and Radiology, Rajas Dental College and Hospital, and then they were assessed for pattern of entry of mental nerve and for the presence or absence of anterior loop of mental nerve. Specific selection criteria were followed considering the impact of gender, age, and dentition on the position of mental foramina. Only the panoramic images of the dentate mandible of patients aged above 18 years were included in the study. History of any traumatic, cystic or tumour lesions involving the inferior alveolar nerve, skeletal deformities, or bony diseases, pathological lesions involving mental foramen area, previous surgery or trauma to the mandible were considered as exclusion criteria. In addition, only the patients whose panoramic radiographs were available in the oral medicine department and were in high-quality images and accuracy were included in the study.

**Figure 1:**
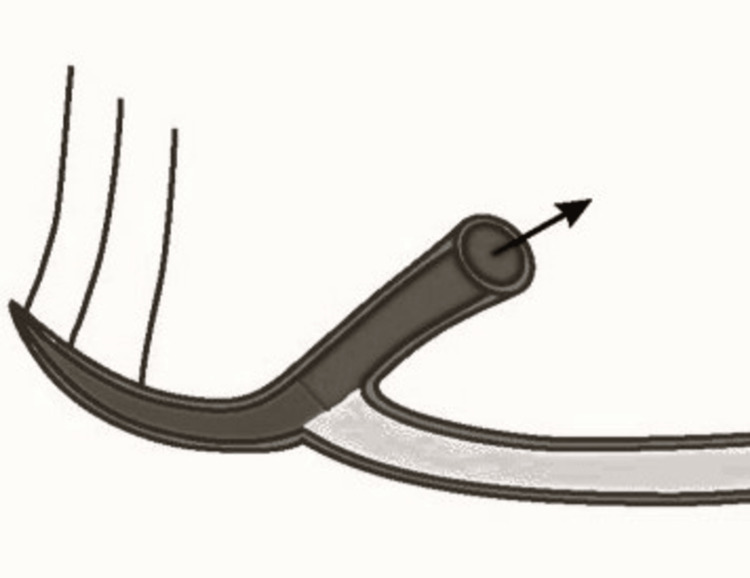
Pictorial representation of anterior loop of mental nerve Picture credit: Divakar Thiruvenkata Krishnan

**Figure 2:**
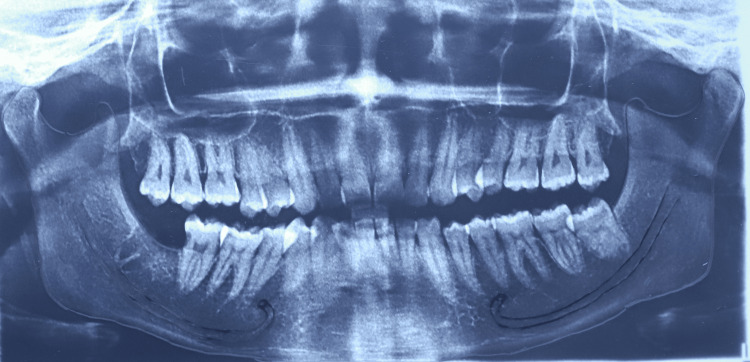
Tracing of panoramic radiograph showing anterior loop of mental nerve One of the digital panoramic images included in this study.

**Figure 3:**
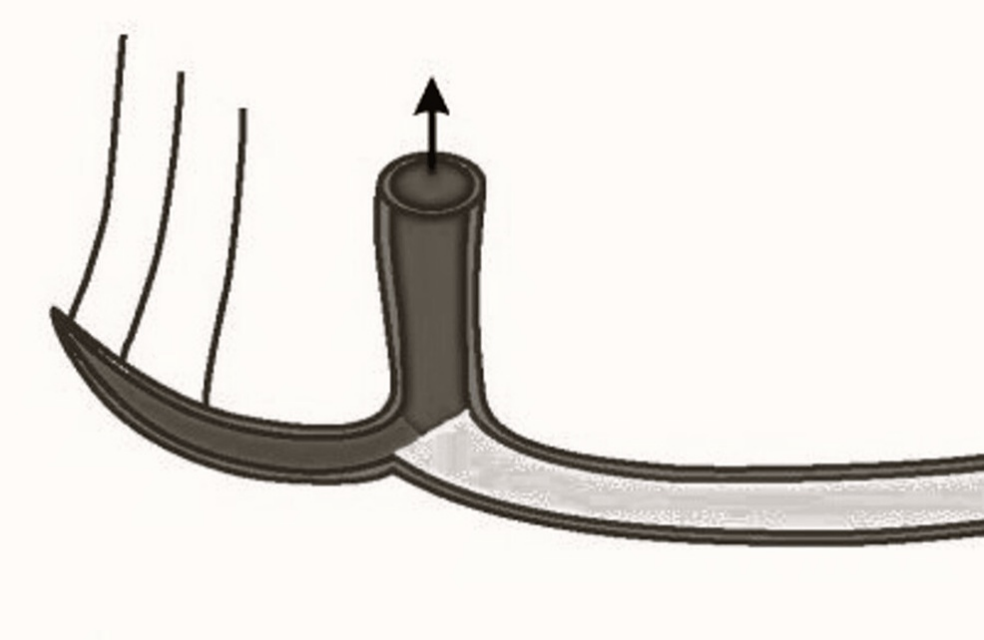
Pictorial representation of the perpendicular pattern of mental nerve Picture credit: Divakar Thiruvenkata Krishnan

**Figure 4:**
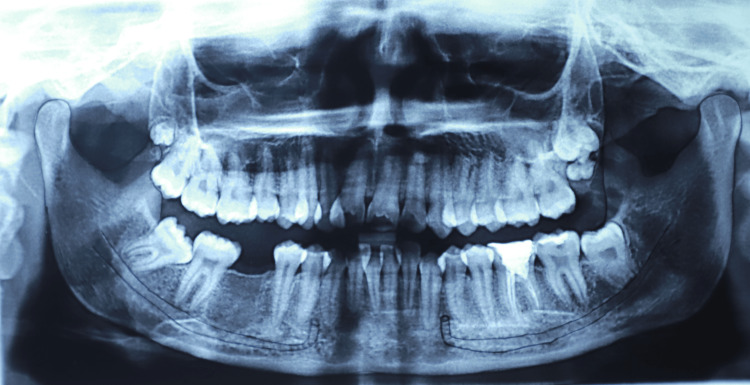
Tracing of panoramic radiograph showing the perpendicular pattern One of the digital panoramic images included in this study.

**Figure 5:**
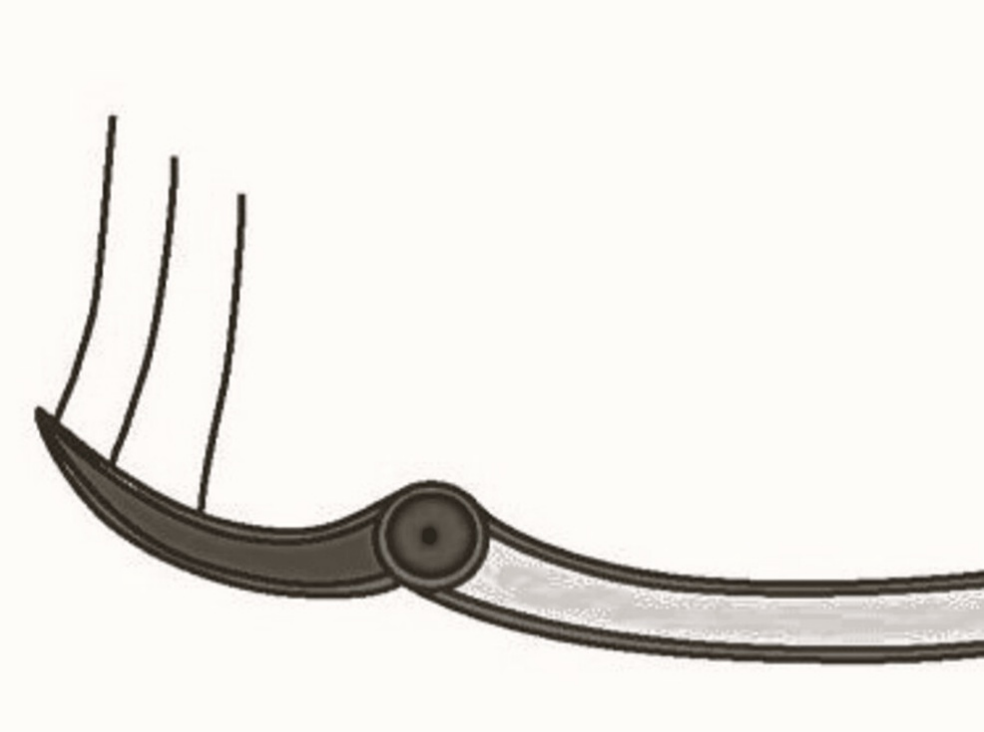
Pictorial representation of the straight pattern of mental nerve Picture credit: Divakar Thiruvenkata Krishnan

**Figure 6:**
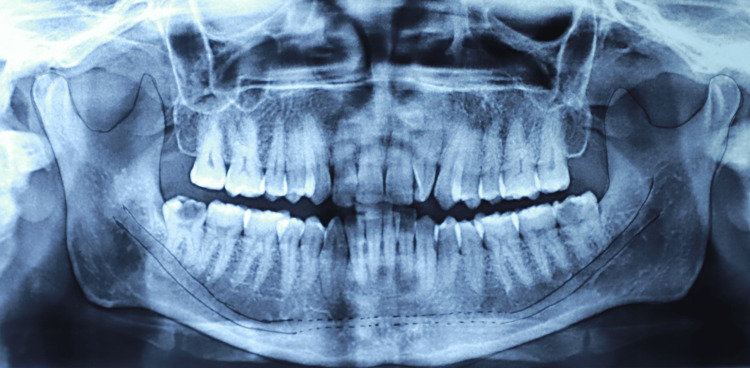
Tracing of panoramic radiograph showing the straight pattern One of the digital panoramic images included in this study.

With agreement between the two independent reviewers, 87 orthopantomogram (OPG) were excluded based on the exclusion criterions mentioned. The frequency and percentage of presence or absence of loop and the pattern of loop were assessed and the results were compared. Any discrepancies in the observation were resolved by a third observer. The different patterns of loop are given below which includes a) Anterior loop pattern (Figures 1, 2) b) Perpendicular pattern and (Figures 3, 4) and c) Straight pattern (Figures 5, 6).

Statistical analysis

Descriptive statistical analyses were performed to assess the prevalence of the pattern of mental nerve. Association between gender and loop pattern and between the sides and subtypes of loop pattern were assessed by Chi-square test. A p-value of less than 0.05 was considered to be statistically significant. All statistical analyses were performed using SPSS software, version 26.0 (IBM Corp., Armonk, NY).

## Results

A total of 300 participants including 600 hemimandibles were evaluated. There was an almost equal gender distribution of participants with 51% females and 49% males. The average age group of the participants were 35.29±10.14 years ranging from 19 to 61 years (Table 1).

**Table 1:** Demographic characteristics of the participants

Demographic factor	Subgroup	Number	Percentage
Gender	Male	153	51.0
	Female	147	49.0
Age	18 -35	172	57.3
	Above 35	128	42.7

In the present study, the most prevalent looping pattern of mental nerve observed was the straight pattern (67.5%) followed by the anterior loop pattern (18.8%) and the perpendicular pattern (13.7%) (Table 2). 

**Table 2:** Frequency of the three observed patterns on 600 hemimandibles

Looping pattern	Frequency	Percentage
Anterior loop	113	18.8
Perpendicular pattern	82	13.7
Straight pattern	405	67.5

The association between the sides, the right and left and the looping pattern statistically analysed by Chi-square test shows there is a highly significant association (Chi-square value=282.193, df=4, p<0.0001) between the right and left side on the looping pattern. The distribution of different looping patterns on both the sides are given in Table 3. The most frequent pattern on both sides were straight pattern with 73% on the right side and 62% on the left side. The frequency of the anterior loop was higher on the left side (23.7%) when compared to the right side (14%) (Table 3).

**Table 3:** Frequency of the three observed patterns on the right and left hemimandibles

Looping pattern	Right side		Left side		P value
	Frequency	Percentage	Frequency	Percentage	
Anterior loop	42	14.0	71	23.7	<0.0001
Perpendicular pattern	39	13.0	43	14.3	
Straight pattern	219	73.0	186	62.0	

The association between the gender vs subtypes of looping pattern on the left and right side by Chi-square test shows there is no significant (p=0.239 and p=0.203 respectively) association between gender and the loop pattern on both left and right side. There is a higher frequency of anterior loop in males (R-15.67%, L-27.25%) when compared to females (R-12.24%, L-19.73%) in both right and left side with the difference being more pronounced on the left side (Table 4).

**Table 4:** Gender distribution of the frequency of the three observed patterns on the right and left hemimandibles

Looping pattern	Right side		P value	Left side		P value
	Male (N = 153)	Female (N = 147)		Male (N = 153)	Female (N = 147)	
	Frequency (%)	Frequency (%)		Frequency (%)	Frequency (%)	
Anterior loop	24 (15.67%)	18 (12.24%)	0.203	42 (27.45%)	29 (19.73%)	0.239
Perpendicular pattern	15 (9.8%)	24 (16.33%)		19 (12.42%)	24 (16.33%)	
Straight pattern	114 (74.51%)	105 (71.43%)		92 (60.13%)	94 (63.94%)	

## Discussion

The inferior alveolar nerve supplies all the mandibular teeth, and its terminal branches are the incisive nerve, the one which runs inside the canal, and the mental nerve, the one which leaves the mental foramen and supplies sensation to the lower lip, buccal mucosa in front of the mental nerve and the skin of the chin ventral to the mental foramen [16]. The intraosseous course of mental nerve has been a debated subject for longer period in the literature but more of it in the point of theoretical analysis (Table 5).

**Table 5:** Global Studies conducted in assessing the looping pattern of the mental nerve since 2013

Sno	Type of study	Result	Sample size	Place of study	Author
1	Radiographic study of mental nerve looping pattern	Anterior loop- 21% Straight- 79% Perpendicular-6%	300	India	(Iyengar et al., 2013) [4]
2	Helical computed tomography scan study to assess anterior loop of mental nerve, mental foramina and incisive nerve	Anterior loop- 53.7%	41	Spain	(Prados-Frutos et al. 2017) [17]
3	Radiographic study of the anterior looping pattern of mental nerve	Anterior loop- 33.5%	272	Bangladesh	(Mojumder et al., 2020) [18]
4	Prevalence of anterior looping pattern of mental nerve using Cone Beam Computed Tomography	Anterior loop- 19%	126	Pakistan	(Rahim et al., 2021) [19]
5	Cone Beam Computed Tomography evaluation of anterior loop length and mental foramen	Anterior loop- 92.9%	112	Vietnam	(Nguyen et al., 2021) [20]

Recent trend of dental implant placement has now kindled the assessment of mental nerve pattern before leaving the foramen for precise implant treatment planning and placement. The interforaminal area which is the most favourable area for dental implant placement is normally believed to be safe area for implant placement because of sparse anatomically important nerves and vessels. The identification of anterior loop of mental nerve has gained anatomical importance because of its clnical relevance [21].

Kieser et al. studied various paterns of loop of mental nerve in cadavers, but there is very less literature available for radiographic studies that have been done so far [22]. The association between the gender versus loop pattern and the association between the left and right sides with the loop pattern hasn’t been studied in the South Indian population in the past studies in the literature. Hence this study aims to determine the prevalence of anterior loop and other patterns (straight and perpendicular) and their association with gender and sides.

The descriptive analysis of the study shows the straight pattern of mental nerve is the most common one which totals (67.5%) followed by anterior loop pattern (18.8%) and perpendicular pattern (13.7%) (Table 2). These results are in accordance with the results of the study conducted by Iyengar et al. (2013), which assessed 300 panoramic radiographs and found the most common pattern as straight followed by anterior loop and perpendicular pattern [4]. Hence there is a 19% chance of occurrence of anterior loop of mental nerve before the emergence of mental nerve from the mental foramen and hence it should be considered while planning for implant placement in the interforaminal and the premolar region. Also, studies have shown that in case of the presence of an anterior loop, there are higher chances that the mental foramen is at a higher level in the alveolar bone which is again a clinically important finding in placing the implant in the premolar region of the mandible [23,24].

The incidence of anterior loop pattern in the current study was 18.8%, with 14% on the right side and 23.7% on the left side. There are varying results reported for the incidence of anterior loop across India including 9.7% in the Eastern Indian population [25], 53.13% in a study carried out in western India [26], 56% in a CBCT based study conducted on Indian population [7]. With regards to the studies conducted in countries other than India, the incidence reported by the current study is higher than those by Jacobs et al. (11%) [27], Arzourman et al. (12%) [1], Yosue and Brooks (21%) [28]. The higher percentage may be due to high resolution of the panoramic images used in this study because of recent advanced device. The difficulty in assessing the looping pattern may be attributed to poor radiograph, poor bone quality and inability to distinguish this structure from trabecular pattern [5]. The incidence of looping pattern also decreases with age as the calcification of cortical bone decreases and the bone repair and remodelling capacity decrease with the age [29]. To overcome this, only dentate mandible has been included in this study because the visualisation and course of anterior loop have been reported to become variable with alveolar resorption after the loss of posterior teeth [22].

The results of the current study also show that there was no significant association between the gender and subtypes of looping pattern and a significant association between side of the mandible and subtypes of looping pattern by Chi-square test (Table 4). This result suggests that the gender has no influence on the occurrence of any of the loop pattern on either side of the jaw. This is similar to the results by Puri et al. [26], who found a lack of sexual dimorphism in the prevalence of anterior loop and in contrast to the study by Gupta et al. [7], and Jena et al. [8], who found a significant association between the prevalence of anterior loop and gender. With regard to the difference in the prevalence of the loop pattern to the side of the mandible, our results are contradictory to Gupta et al. [7] and Puri et al. [26], who did not find a significant association between the side of the mandible and prevalence of anterior loop and similar to the study by Jena et al. [8] who found a higher prevalence of anterior loop in males and on the left side. This shows that the looping pattern of mental nerve exhibit variations in terms of gender and side prevalence among the Indian population.

The systematic review by Pele et al. [30] to assess whether the anatomy of mental foramen is precisely evaluable with cone beam computed tomography (CBCT) before implantation in humans suggests certain limitations of two-dimensional imaging, such as distortion, magnification, and superimposition. This limitations have been overcome by using CBCT-reformatted panoramic images over panoramic images in identifying the mandibular canal because these images are free of magnification and superimposition and better in detecting anatomical particularities of each patient and supported the use of CBCT to evaluate the patient anatomy to avoid nerve injury before surgery. Many studies suggest that panoramic radiographs are limited in their ability to visualize the anterior loop as failure to view loop in panoramic radiograph doesn’t mean it’s absent [1,5,28]. Hence within the limitations of this study it is concluded that the anterior loop pattern of mental nerve has been found in 18.8% of the population suggesting to accurate planning with three dimensional imaging techniques to avoid the nerve injury to mental nerve during dental implant placement and other surgical procedure involving the interforaminal region of the mandible.

The limitation of the study is because of panoramic radiograph used for assessment is of two dimensional and has magnification errors when compared to more accurate CBCT and CT scans. Within these limitations the results the study concludes that straight pattern of mental nerve is most common followed by anterior loop and perpendicular pattern.

## Conclusions

The knowledge of course of the inferior alveolar nerve and the variation in the looping pattern of mental nerve plays a pivotal role for planning in Implantology. The post-operative paraesthesia in interforaminal implant placement is most commonly due to the violation of the canal or into the loop. The prevalence and frequency of nerve pattern and anterior looping of mental nerve widely varies with different population groups. This study retrospectively, access the prevalence and the pattern of the anterior loop in South Indian population, by using panoramic radiograph. The pattern of the mental nerve plays a vital role in implant placement. Within the limitations of the study, it is suggested to have accurate planning and diagnosis is required to prevent the nerve injury in the region of mental foramen as it is not common to have anterior loop and other pattern which may get injured during dental implant placement and in other surgical procedures. Further studies with higher imaging modalities may be required to accurately predict the presence of a pattern of mental nerve to prevent its injury from any surgical procedure in the premolar region.
